# Ethnic Differences in Barriers and Enablers to Physical Activity Among Older Adults

**DOI:** 10.3389/fpubh.2021.691851

**Published:** 2021-09-10

**Authors:** Emily You, Nicola T. Lautenschlager, Ching Shan Wan, Anita M. Y. Goh, Eleanor Curran, Terence W. H. Chong, Kaarin J. Anstey, Fahad Hanna, Kathryn A. Ellis

**Affiliations:** ^1^Academic Unit for Psychiatry of Old Age, Department of Psychiatry, The University of Melbourne, Melbourne, VIC, Australia; ^2^NorthWestern Mental Health, Royal Melbourne Hospital, Melbourne, VIC, Australia; ^3^Department of Medicine and Aged Care, @AgeMelbourne, The Royal Melbourne Hospital, The University of Melbourne, Parkville, VIC, Australia; ^4^Nursing Research Institute, St. Vincent's Health Network Sydney, St. Vincent's Hospital Melbourne, Australian Catholic University, Melbourne, VIC, Australia; ^5^National Ageing Research Institute, Parkville, VIC, Australia; ^6^Department of Psychiatry, Melbourne Neuropsychiatry Centre, The University of Melbourne, Melbourne, VIC, Australia; ^7^St. Vincent's Hospital, Melbourne, VIC, Australia; ^8^The University of New South Wales (UNSW) Ageing Futures Institute, University of New South Wales, Kensington, NSW, Australia; ^9^Neuroscience Research Australia, Randwick, NSW, Australia; ^10^Program of Public Health, Torrens University Australia, Melbourne, VIC, Australia; ^11^Higher Education College, Chisholm Institute, Dandenong, VIC, Australia; ^12^Melbourne School of Psychological Sciences, University of Melbourne, Melbourne, VIC, Australia

**Keywords:** barriers, enablers, ethnicity, older age, physical activity

## Abstract

Despite its well-known health benefits, most older adults do not commit to undertaking sufficient physical activity (PA). In this study we aimed to examine the perceived benefits of and barriers and enablers to PA from the perspectives of older Caucasian and Chinese adults living in Australia. Individual and group interviews with 17 Caucasian (mean age: 72.8 years) and 47 Chinese adults (mean age: 74.0 years) were conducted and analysed using thematic analysis. Overall, participants knew about the benefits of PA on physical health but had inconsistent views on its benefits on mental and cognitive health. Older Caucasian and Chinese adults reported similar barriers (e.g., health issues, costs, bad weather and lack of time) and enablers (e.g., improving health; environmental enablers such as adequate and walkable spaces and good natural environment; peer support; and self-motivation) to PA. In comparison, older Chinese adults reported barriers more often, and reported some unique barriers relating to language and culture issues. The findings contribute to developing targeted PA programs for older Caucasian and Chinese adults.

## Introduction

Globally, physical inactivity is one of the top ten risk factors for disease burden and contributes to 9% of all deaths (1). Evidence suggests that physical activity (PA) is associated with physical, psychological and cognitive benefits across the life span, including older age (1–4). However, the majority of older adults do not engage in recommended levels of PA (1). Moreover, older adults tend to undertake less PA with advancing age (5).

Australian data show that 75% of Australians aged 65 years and older are insufficiently active (<150 min of moderate PA/week). This increases to 79% of men and 89% of women aged 85 years and over, respectively (6). There is disparity in PA between Asian and Caucasian adults in western countries. Research from the United States (US) of America reported that older Asian adults were less likely to participate in PA compared with older Caucasian, Latino and African Americans, and, older Chinese immigrants were less physically active compared with older Vietnamese and Korean immigrants (7). While there is a lack of comparative research in the Australian context, one study reported that older Chinese adults in Australia had very limited participation in PA (8).

“Physical activity is defined as any bodily movement produced by skeletal muscles that results in energy expenditure… Physical activity in daily life can be categorised into occupational, sports, conditioning, household, or other activities (9).” Extensive research has examined barriers and enablers to PA in older adults (1, 10, 11). However, research on this topic involving a direct comparison between older ethnic groups (such as older Chinese) and older Caucasians is rare. We identified one US study comparing barriers and enablers to PA among Caucasians, Chinese, Vietnamese, African Americans, American Indians and Latinos (12). The findings showed that common barriers to PA in older Caucasians and older ethnic minority groups were health issues, fear of falls and inconvenience, while common enablers included positive outcome expectations, health benefits, social support and access to PA programs. This study also found differences in perceived barriers and enablers among those ethnic groups. For example, overdoing exercise and feeling too old were barriers, and medical assistive devices (e.g., a walker) and low-cost activities were enablers for Caucasians. Inconvenient built environment and lack of knowledge about PA were barriers, and health benefits were an enabler for American Indians. Lastly, lack of time was a barrier for African Americans and Vietnamese immigrants (12).

Australia is a culturally diverse country where the Chinese population (1.2 million; ~6% of the total population) is one of the largest and fastest-growing ethnic minority groups (13). Within the Chinese population individuals may have different countries of origin (e.g., China, Singapore, Malaysia and Vietnam); however, they share common cultural heritage and may hold similar values and beliefs about culturally acceptable behaviours (14). These values and beliefs might affect their lifestyle, attitudes and behaviours after immigrating to a new country (14). Giving the importance of understanding and reducing health disparities in Australia, it is important to identify factors that hinder and promote PA in the older Chinese population and compare the differences in these factors between this large ethnic minority group and older Caucasians.

To our knowledge, to date no research has been published that compares barriers and enablers to PA between older Caucasian and Chinese adults living in Australia. Such research would provide evidence to support the development of culturally appropriate PA programs facilitating older adults from different ethnic groups to engage in and benefit from PA. This research would further contribute to reducing public health expenditures associated with chronic diseases caused and exacerbated by physical inactivity.

In this qualitative study we aimed to examine and compare perceived benefits of and barriers and enablers to PA in older Caucasian and Chinese adults living in Australia. The research questions are: (1) What are the benefits of PA perceived by older adults? (2) What are the barriers and enablers to PA in older adults? (3) Are there similar and different views on the barriers and enablers to PA between older Caucasian and Chinese adults living in Australia?

## Materials and Methods

### Theoretical Framework

The Social-Ecological Model (SEM) provides a multi-level factors model which typically focuses on individual, interpersonal, environmental and policy factors. Individual factors are related to individuals' characteristics, experiences, knowledge, attitudes, beliefs and skills. Interpersonal factors refer to individuals' social networks and support, and cultural practises. Environmental factors include the weather, and neighbourhood environments such as infrastructure, parks, public transport and safety etc. Lastly, policy factors include guidelines, policies, laws, regulations etc. (15, 16). This model has been widely used to understand the influences of these multi-level factors on PA (15–17). In this study, this model was used to guide the reporting and discussion of the findings relating to multi-levels of barriers and enablers to PA from the perspectives of older Caucasian and Chinese adults. The use of this model also enabled us to make meaningful comparison regarding those multi-levels of barriers and enablers between the two ethnic groups.

### Study Design

In this qualitative study we employed a phenomenological approach which usually seeks to explore a phenomenon (namely barriers and enablers to PA in this study) from the perspectives of those (namely older Caucasian and Chinese adults in this study) who experience it (18). The use of this approach allowed us to gain an in-depth understanding of a social phenomenon experienced by individuals themselves (19).

### Ethical Approval and Consent

Ethical approval was obtained from the Human Research Ethics Committee of the University of Melbourne (Ethics ID: 1750807). We provided each participant with a plain language statement, project flyer and consent form written in English. We communicated the project information to participants in Mandarin or Cantonese verbally, if required. Most participants provided written consent while nine Chinese participants provided verbal consent for cultural reasons. The verbal consent was audio recorded at the beginning of the interview.

### Participants

Participants were community-dwelling, older (≥60 years) Caucasian and Chinese adults living in Australia. Chinese people in this context were defined as of Chinese ancestry, self-identifying as Chinese, and/or speaking Mandarin or Cantonese as a first language.

Exclusion criteria reflected characteristics that may significantly influence an individual's ability or motivation to undertake PA. These included self-reported or diagnosed severe medical conditions, such as physical disability, heart disease, stroke, psychosis, major depression, dementia, Parkinson's disease and progressive malignancy.

### Sampling and Recruitment

To capture a wide range of views, we used a purposive sampling strategy and considered a number of participant characteristics when recruiting participants. These factors were age, gender, body mass index (BMI), education, marital status, location, years of living in Australia, self-reported health, and self-reported PA level.

Our recruitment strategies included advertising the project on the websites of relevant research institutes and community organisations; using Twitter, Facebook and LinkedIn; seeking assistance from senior clubs, community organisations, libraries, news agencies and radio stations at National and State levels; and giving community talks.

### Data Collection

We conducted semi-structured interviews with participants between March and November 2018. The sample size was determined using the data saturation principle, meaning that data collection ceased when no new information emerged from new interviews (20). We employed different interview methods based on our experience and the literature (21) and older adults' preferences (individual vs. group interviews and face-to-face vs. telephone interviews). Specifically, we conducted individual interviews with older Caucasians at their homes or *via* phone in English. For older Chinese adults, we conducted individual interviews *via* phone, and group interviews at participants' homes or community centres. We used English, Mandarin or Cantonese to interview Chinese participants. EY and CW, both were bilingual researchers, undertook 11 individual interviews with Caucasians and 1 group interview with Chinese adults together in English. For logistic reasons, they conducted the other individual or group interviews independently using English, Mandarin and/or Cantonese.

The duration ranged from 16 to 69 min for individual interviews and from 45 to 60 min for group interviews. We collected information on participant characteristics (Table 1) prior to or during the interviews. We used the same interview guide to conduct individual and group interviews (see Appendix).

**Table 1:** Participant characteristics.

**Participant characteristics**	**Caucasians (*N* = 17)**	**Chinese (*n* = 47)**	**Total**
	**Mean (SD)**	**Mean (SD)**	**Mean (SD)**
Age	72.8 (5.3)	74.0 (8.5)	73.7 (7.8)
Years of education	16.0 (4.1)	10.8 (4.9)	12.2 (5.2)
Immigration age		48.6 (17.4)	
Years living in Australia		25.7 (13.7)	
	***n*** **(%)**	***n*** **(%)**	***n*** **(%)**
Gender (male)	10 (41.2)	14 (36.2)	24 (37.5)
Birthplace			
Australia	15 (88.2)		
UK	2 (11.8)		
China (including Hong Kong and Taiwan)		33 (70.2)	
Other (Malaysia, Vietnam etc.)		14 (29.8)	
BMI categories			
Underweight		1 (2.3)	1 (1.6)
Normal	13 (76.5)	30 (68.2)	43 (70.5)
Overweight	3 (17.6)	12 (27.3)	15 (24.6)
Obese	1 (5.9)	1 (2.3)	2 (3.3)
Marriage status			
Single		3 (6.4)	3 (4.7)
Married	13 (76.5)	20 (63.8)	43 (67.2)
Divorced	1 (5.9)	2 (4.3)	3 (4.7)
Widowed	1 (5.9)	11 (23.4)	12 (18.8)
Cohabiting/defacto	2 (11.8)	1 (2.1)	3 (4.7)
Place of current living			
Suburb	16 (94.1)	47 (100)	63 (98.4)
Rural	1 (5.9)		1 (1.6)
Self-rated general health			
Poor		6 (12.8)	6 (9.4)
Fair	3 (17.6)	26 (55.3)	29 (45.3)
Good	14 (82.4)	15 (31.9)	29 (45.3)
Self-rated as being active			
Inactive		6 (12.8)	6 (9.4)
Less active	3 (17.6)	25 (53.2)	28 (43.8)
Active	14 (82.4)	16 (34.0)	30 (46.9)

We audio-recorded all interviews with participants' consent. Professional transcriptionists transcribed all English interviews verbatim. CW transcribed the Chinese interviews and EY doubled checked the accuracy of the transcripts. The interview transcripts provided the primary data source which was supplemented by field notes taken during the interviews.

### Data Analysis

We analysed the data at the interview level (each group interview was treated as an individual interview) using thematic analysis (22). This data analysis method is appropriate as it has been applied in previously published research examining barriers and enablers to PA (12, 22). In addition, thematic analysis is a flexible qualitative analytical method, which allows us to determine themes (and frequency/prevalence) in a consistent way when analysing the similar and different views between older Caucasian and Chinese adults (22). Our data analysis involved four, iterative stages: familiarising with the data through reading and re-reading each transcript; generating codes using words, phrases or short sentences; identifying themes and subthemes based on the codes; and reviewing and finalising the themes and subthemes (22).

We used QSR NVivo 11 to store, organise, retrieve and code the data. EY coded the data using an inductive approach and identified potential themes and subthemes concerning the benefits of and barriers and enablers to PA. All co-authors interpreted the themes and subthemes until agreement was reached. We reported the findings according to the themes and subthemes and used exemplar quotes provided by Caucasian (CAU + number) and/or Chinese (CHI + number) participants to support the findings. Where necessary we provided additional information in brackets to ensure the clarity of the quotes.

## Results

We interviewed in total 64 Caucasian and Chinese adults. These included 17 individual interviews with Caucasians, 10 individual interviews with Chinese adults, and 5 group interviews with 37 Chinese adults. As mentioned above, each group interview was treated as an individual interview for the purpose of data analysis—identifying themes and subthemes.

The mean age of the whole study sample was around 74 years (7.8 SD) and the majority were women (62.5%). Compared with Chinese adults, Caucasians on average received more years of education (16 vs. 11 years). Furthermore, higher proportions of Caucasians were married (76.5 vs. 63.8%), self-rated as physically active (82.4 vs. 34.0%), and rated their health as good (82.4 vs. 31.9%) (see Table 1).

Across the interviews, participants used the terms PA and exercise interchangeably. Most participants interpreted PA as a type of body movement that could be structured or unstructured, and gentle or vigorous. Examples of PA included housework, gardening, walking, running, swimming, cycling, yoga, Tai Chi, dancing, Pilates, gym exercise and playing sports. A few participants did not regard housework, gardening or slow walking as PA due to their low level of intensity.

Themes and subthemes relating to perceived benefits of PA and multi-level barriers and enablers to PA are described below (see further details in Table 2).

**Table 2:** Benefits of and barriers and enablers to PA in older Caucasian and Chinese adults.

**Themes and subthemesNote: where relevant, subthemes are mapped to the components of Socio-Ecologic Model (SEM)**	**Caucasians (CAU) (*n* = 17)**	Chinese (CHI) (*n* = 15)^**^	**Themes/subthemes reported more often by older CAU or CHI adults**
	**Themes/subthemes reported (Yes/No)**		
**Theme 1. Perceived PA benefits**			
Physical health and functioning	Yes	Yes	
Mental health	Yes	Yes	
Brain health	Yes	Yes	
**Theme 2. Barriers**			
**2.1 Key Barriers (reported very often)**			
SEM—Individual related barriers			
Health issues	Yes	Yes	
Lack of time	Yes	Yes	CHI
Lack of motivation	Yes	Yes	CHI
Older age	Yes	Yes	
Concerns about injuries and harms	Yes	Yes	
Lack of interest	Yes	Yes	CHI
SEM—Individual and interpersonal related barriers			
Language and culture-related barriers: lack of fluency in English, feeling reserved, and concerns about inconveniencing people	No	Yes	
SEM—Environment related barriers			
Cost concerns (affordability)	Yes	Yes	CHI
Bad weather	Yes	Yes	
Distant exercise location	Yes	Yes	CHI
Other environment related barriers	Yes	Yes	CHI
**2.2 Barriers reported less often (in** ** <10 interviews)**			
SEM—Individual related barriers			
Feeling tired: *Sometimes, yeah, I just might feel tired and don't feel able to do it. CAU014*.	Yes	Yes	
Difficulty and complexity of the exercise/Individual ability: *In the gym the exercise is too hard, too heavy for me like walking on the treadmill and all those sort of things you know. I just prefer to take a walk. CHI006*	Yes	Yes	CHI
Lack of knowledge: *I couldn't do yoga but it's really more that you don't know what yoga's about. CAU011*.	Yes	Yes	
Lack of confidence: *There will be people who aren't as self-confident who probably don't go out because they'll feel embarrassed that they're walking slowly or can't walk or have pain and the like. CAU007*.	Yes	Yes	
SEM—Interpersonal related barriers			
Lack of family or peer support: *Family, I'd be really pleased if my husband would help me a bit more and I might do more exercise. CAU009*.	Yes	Yes	
SEM—Environment related barriers			
Anti-exercise environment: *You wouldn't be too inspired to get up and do anything would you if everybody else around you just sat and watched television or whatever. CAU011*.	Yes	Yes	
**Theme 3. Enablers**			
**3.1 Key enablers (reported very often)**			
SEM—Individual related enablers			
Maintaining or improving health	Yes	Yes	
Self-motivation	Yes	Yes	CAU
Enjoyment	Yes	Yes	CAU
Maintaining a habit (partially overlapping with previous exercise experience)	Yes	Yes	CAU
Having a fulfilling life	Yes	Yes	CAU
Having more time after retirement	Yes	Yes	
SEM—Interpersonal related enablers			
Peer influence or support	Yes	Yes	CHI
Social aspects of exercise	Yes	Yes	CAU
SEM—Environment related enablers			
Common environment related enablers	Yes	Yes	
Pro-exercise culture	Yes	Yes	CHI
**3.2 Enablers reported less often (in** ** <10 interviews)**			
SEM—Individual related enablers			
Goal setting: *I'm not aiming to run 6 kilometres a day or something, I'm not aiming to do that, I'm just aiming to very, very slowly increase my stamina, I'm already pleased with the amount that I do. CAU013*.	Yes	Yes	CAU
Having a pet: *The dog is used to the routine and so she will come to me and say, in her way, come on, time to go, and I dare not go because I care about her too so we're good for each other. CAU016*.	Yes	Yes	CAU
Use of technology: *I have a Fit Bit and I try and do my 10,000 steps a day – don't always succeed but I'm aware that it's very important to keep moving. CAU010*.	Yes	Yes	
Self-discipline: *Exercise first that's my priority. Exercise first – everything else will come up to that and then you can plan your time for the other activities. CHI007*.	Yes	Yes	
SEM—Interpersonal related enablers			
Unwilling to become a burden on children: *I do exercise for keeping healthy and not becoming a burden to my children. If I were sick, I would cause trouble to my husband, sons and daughter-in law. CHI013*.	No	Yes	
People's compliment: *I think to a certain extent, what is a driving force is vanity, I'm embarrassed to say this, because you know every time someone says “How old are you, oh no, oh you look terrific,” that's wonderful for me. CAU003*.	Yes	Yes	
SEM—Community or organisation related enablers			
PA opportunities: *If there are more group activities (provided by this Chinese association) we can walk more … we feel even happier if we go out, we can walk more. CHI003*.	No	Yes	
**Themes 4. Support of the health system, community and government to do PA (barriers and enablers)**			
GP support (SEM—Community or organisational factors)	Yes	Yes	
Community support (SEM—Community factors)	Yes	Yes	
Government support (SEM—Policy factors)	Yes	Yes	

1) ^**^47 Chinese participants attended five group interviews and 10 individual interviews. Each focus group was treated as an individual interview for the data analysis purpose.

*2) GP, general practitioners*.

*3) Representative quotes are only provided for those barriers and enablers less often reported by participants in the table*.

### Perceived Benefits of PA

Participants' understanding of the benefits of PA centred on physical, mental, and brain health. Some participants also commented on its benefits on overall health and well-being, and sleep.

### Physical Health and Functioning

According to participants, PA had numerous benefits for physical health and functioning. These included improving heart function, body movement, balance, and flexibility; reducing the risk of falls and developing chronic diseases; strengthening muscles and bones; relieving pains; and controlling weight. PA could also improve some medical conditions, such as stroke, heart disease and diabetes. As one participant expressed: “I had a stroke 10 years ago and half of my body could not move. So I started to do exercise, Tai Chi.” CHI003.

Some participants commented that PA could improve health in general, such as feeling healthier and living longer: “You find that older people who walk or exercise a lot, they live longer lives.” CHI001. Others thought PA could improve sleep: “I think that goes hand in hand with the exercise too—is having good sleep and that certainly is beneficial too.” CAU010.

### Mental Health

Participants related mental health to feeling happier or having a good mood, and believed that PA could benefit mental health. A few participants specifically mentioned that PA could improve depressive symptoms: “I found that if I walked, walked for several thousand steps every day, when I walked in the morning, I could gradually adjust my thoughts and I won't have the depressive symptoms.” CHI014. When exploring this topic deeply, we found that most participants attributed the mental health benefits to the social aspect of PA rather than PA itself: “So, exercise, so it's physical, but mentally, on the other side, you benefit as well, because you've got friends, you've got a bigger social circle …” CHI016.

### Brain Health

Participants had mixed views about the brain health benefits of PA. Some participants reported that PA stimulated the brain to think or helped with memory: “I think it stimulates the brain a bit, yeah. Because especially with the dancing you have to remember the steps …” CHI006. A few participants believed that PA could reduce the risk of developing dementia:

It certainly will have a lot – help the whole lot – keep away the dementia side of things and it's no good just doing it (exercise) once a month, it's no good just doing it once a week, you've got to have a bit of a … nearly every day. CAU015

Some participants imagined that PA was beneficial to brain health: “I would imagine it would, don't ask me how, I'm not a doctor, but I would think it's beneficial at every level. I'm sure it helps the brain, I mean, just at a practical level. It would help problem solving.” CAU017. Some participants were not sure:[Interviewer: How about cognitive health?]. “Oh, I'm not so sure about that.” CAU004. Other participants were sceptical about the brain health benefits of PA: “The brain … I can't feel it … I don't feel it.” CHI001.

### Perceived Barriers and Enablers to PA

We identified specific barriers and enablers to PA (subthemes) and mapped them to individual, interpersonal, environmental, community or organisational, and policy factors according to the Social-Ecological Model (SEM) (see Table 2).

As shown in Table 2, only older Chinese adults reported language and culture related barriers (individual and interpersonal related barriers), unwilling to burden children (interpersonal related barrier) and PA opportunities (community or organisation related enabler). While both older Caucasian and Chinese adults reported the other barriers and enablers listed in the table, older Chinese adults more often reported some individual related barriers (lack of time, lack of motivation and lack of interest), environment related barriers (cost concerns, distant exercise location and other environment related barriers), interpersonal related enabler (peer influence or support) and environment related enabler (pro-exercise culture). In contrast, older Caucasians more often reported some individual related enablers (self-motivation, enjoyment, maintaining a habit, and having a fulfilling life), and interpersonal related enabler (social aspects of exercise).

The key barriers and enablers to PA reported by older Caucasian and Chinese adults are described below. Unless clearly specified through the comparison of the findings, older Caucasian and Chinese adults' views on the barriers and enablers were identified as similar.

### Key Barriers to PA

#### SEM—Individual Related Barriers

##### Health Issues

Most participants from both groups reported having medical issues (e.g., diabetes, arthritis and pains.), which affected their ability to undertake or maintain PA: “If I have pain I would sort of be reluctant to do exercise … that is probably the main barrier for me.” CHI001.

##### Lack of Time

Family duties, such as childcare and housework, were barriers to PA. Compared with older Caucasians, older Chinese adults more often reported that caring for grandchildren, part of the Chinese culture, left them no time to do exercise: “For Chinese people in our age, how couldn't we help? As parents, we are making sacrifice for caring for grandchildren. In doing so we really do not have our own time.” CHI003. In contrast, some older Caucasians had different opinions on the impact of family duties on PA. First, doing housework and caring for grandchildren involved PA: “I mean I've got eight grandchildren. I do a lot of exercise when I'm with them, but that's not organised.” CAU008. Second, despite the family duties, they could manage their time to do exercise: “It depends if you can work around it – you can usually work out a schedule of some kind that allows you to get adequate exercise.” CAU012.

##### Lack of Motivation

Lack of motivation was a common barrier for many older adults to do exercise. Some participants reported the lack of motivation to do exercise alone: “Yeah, I haven't got the motivation to really exercise on my own.” CAU002. Some participants reported feeling lazy: “I am lazy, I do not want to do exercise.” CHI010.

##### Older Age

Older age was reported as a barrier to PA: With age older adults felt physically incapable of or lacking confidence in doing exercise: “When I get on my bicycle I do feel quite self-conscious about how old I am compared to everybody else … so I can see sort of thing, as I get older (it) might be a barrier.” CAU013.

##### Concerns About Injuries or Harms

Concerns about injuries or harms presented a barrier to older adults to do exercise or do the same exercise as they had done before: “I used to ride bike. No matter how far away the destination was, I would ride there. Now I do not have the courage, I am worried I would fall over.” CHI003

##### Lack of Interest

Many older adults reported lacking interest to do or maintain specific types of exercise. For example: “I like the walking bit but I find actual exercise really boring, like stretching or weights or something like that.” CAU013.

#### SEM—Individual and Interpersonal Related Barriers

##### Language and Culture-Related Barriers

Compared with older Caucasians, older Chinese adults reported some unique barriers to PA. First, lack of fluency in English inhibited some older Chinese adults to obtain information on PA and access PA opportunities: “They don't know English, so they don't know there's this thing (exercise class) so that they can join.” CHI008. Second, from the cultural perspective, being reserved and concerns about inconveniencing people presented barriers to older Chinese adults to attend exercise or social groups: “There's a culture … You don't want to trouble other people, to inconvenience people. The Europeans are different, they're more open-minded about things, they're more outgoing, you see Italian people go dancing, line dancing, and singing and finding all the clubs for activities …” CHI017.

#### SEM—Environment Related Barriers

##### Cost Concerns

Gym exercise and some group exercise such as golf, dancing and yoga involved costs. Older Caucasians had mixed views about paying for exercise. For example, some Caucasians were concerned about costs and therefore avoided doing exercise involving payment: “It (Tai Chi) might have been $7, whatever it was, I decided not to do it because cost is important, I am a pensioner.” CAU009. Some Caucasians thought it was unnecessary to pay to do exercise even though they could afford it: “I can't think of a reason to go to pay to exercise, there are so many things you can do for (paying) nothing.” CAU003. Other Caucasians were willing to pay: “I can afford it and it is in a pool that has to be maintained by somebody.” CAU017.

In comparison, older Chinese adults more often expressed concerns about the cost of exercise: “If it (Tai Chi) needs to pay, many people will not learn. Older people do not have money.” CHI004. Therefore, many older Chinese adults preferred free exercise such as walking: “Walking is the best, you do not need to pay.” CHI003.

##### Bad Weather

Bad weather, such as being hot, cold, rainy or cloudy, affected older adults' ability, mood or motivation to do exercise: “I guess the weather it prevents you from going out … like going out today when it's cold and wet. You just keep yourself warm at home, so of course you won't exercise when you're home.” CHI006. However, some older adults would still do exercise in bad weather; for example in rainy days some older adults would still go for a walk by wearing appropriate shoes and clothes, and others would do indoor exercise as an alternative.

##### Distant Exercise Location

Distant exercise location was a common barrier to PA for many older Chinese adults who relied on public transport to travel: “If it's the right line for wherever the place you want to go to that's fine, but there are a lot of places you have to change from bus to train, or train to train, or something like that.” CHI010. Some older Caucasians who were still driving regarded it as unnecessary to waste time travelling a long distance to do exercise: “You know, you don't want to be travelling for three-quarters of an hour to get somewhere to be able to start your exercise.” CAU016.

##### Other Environment Related Barriers

Participants raised a few other environment related barriers to PA including the lack of exercise facilities nearby, poor natural environment (e.g., lack of parks, and unsafe or crowded streets.), and neighbourhood safety. For example:

I feel because there are fewer people in Australia (compared with China), so they have fewer exercise facilities. I hope they can provide more facilities. CHI011
We have unmade roads because that's what we want but they're safe because there's not a lot of traffic. That would be one of the barriers to me, if I didn't feel safe I wouldn't do it. CAU017

### Key Enablers to PA

#### SEM—Individual Related Enablers

##### Maintaining or Improving Health

Almost all participants reported that maintaining or improving health (more often relating to physical health or the general health and well-being) motivated them to do exercise: “I want to do it (exercise), I like to feel healthy, feel well.” CAU002. One older Chinese adult reported that her motivation of doing exercise included preventing dementia: “I do exercise for the purpose of preventing this (dementia). If I got dementia, my children would live miserable lives. ”CHI013.

##### Self-Motivation

Self-motivation was an important factor, driving many older adults to do exercise: “The motivation is important, people want to do it (exercise) for themselves, that's all I say ….” CHI017. With self-motivation some older adults could overcome barriers to PA: “I don't feel that there are any barriers to it, no. You just push yourself to go and do what you want to do.” CAU002.

##### Enjoyment

In contrast with the lack of interest as a barrier to PA, enjoyment or interest motivated older adults to do exercise. This factor also affected some older adults' choice of doing or maintaining specific types of PA. For example: “You know I have always just enjoyed that thing about going out and exercising hard and coming home and being tired from exercising.” CAU006.

##### Maintaining a Habit

A strong theme for older Caucasians but not for older Chinese adults was that maintaining a habit (in other words being physically active previously) was a reason why they continued to do exercise. As one participant expressed: “I think if you start exercising as a teenager, then it's really, it's kind of part of your lifestyle, then you, I guess you just keep on going.” CAU004.

##### Having a Fulfilling Life

Participants, in particular older Caucasians reported that having a fulfilling life (e.g., keeping busy, having something to do and enjoying other part of life.) motivated them to do exercise. For example: “I suppose we (my friend and I) are both of the same mind that, you know, that you've got to sort of keep moving to make sure that you function as well as you can. And then you enjoy life more, that's the whole point.” CAU005.

##### Having More Time After Retirement

In contrast with the lack of time reported as a barrier to PA, having more time after retirement enabled some older adults to do more exercise, choose exercise that was enjoyable, and maintain an exercise routine. For example:

I am a retiree, so I have my set of routine each day. When I get up, I have the time, that certain time that I get up, and I do my exercise, and then I have a shower and whatever. CHI016
I used to do swimming regularly once just because I could but I didn't like it that much … Now because I can choose to do things that are much more enjoyable I wouldn't bother because I haven't got the time worries. CAU005

#### SEM—Interpersonal Related Enablers

##### Support or Influence of Peers

Support or influence of peers (such as partners and friends) facilitated older adults to do exercise. First, peers provided older adults with motivation, support and encouragement to do exercise: “If I have a company, I will have the motivation to play it (Tai Chi).” CHI003. Second, older adults felt the commitment to their peers to do exercise: “I can do that tomorrow or the day after that, but it's a group, you're committed.” CAU011. Third, the opportunity to socialise with peers motivated older adults to do and maintain exercise: “That is part of this group exercise thing as well, it's a friendship. You make friends and get to know people and you're more inclined to keep going.” CAU010.

#### SEM—Environment Related Enablers

##### Common Environment Related Enablers

As opposed to some environment barriers to PA described above, availability of exercise facilities, adequate and walkable spaces, and good natural environment facilitated older adults to do exercise. For example: “When you live near a quiet street and there are parks around, it's a good place to exercise.” CHI008.

##### Pro-exercise Culture

Lastly, a pro-exercise culture (namely community or society's emphasis on PA) facilitated some older adults to do exercise: “I think in our town we created our own facilities, we create our own activities because that's what we are.” CAU002. To this end, some older Chinese adults mentioned the positive societal change from emphasising surviving to living a good life that included doing exercise: “In our generation, due to the poor economy, everyone thought about making a living, we had no time to do exercise… Now the economy has improved, people can change their lives, such as doing exercise to improve their health.” CHI014.

#### SEM—Community or Organisation, and Policy Related Factors: Support of the Health System, Community and Government to Do Exercise

Since participants rarely discussed barriers or enablers to PA at the level of the health system, community or government, we prompted them to think about whether they needed support from these sectors to do PA.

#### GP Support

Participants had mixed views about the role of health care professionals in particular general practitioners (GPs) in supporting older adults to do exercise. First, GPs either provided general advice or had no time to provide advice on PA: “They've only got so many minutes so they really don't have time to promote this sort of thing.” CHI008. Second, it was not necessary to seek advice from GPs: “No, I don't think it's necessary to get that advice from a doctor to exercise.” CAU015. Third, GPs should provide exercise advice because older people saw them very often and trusted them: “They should encourage, because they play an important part, the GP, for the older people.” CHI017.

#### Community and Government Support

Participants commented that community and government should support older adults to participate in PA. This could involve providing information on the benefits of PA and PA opportunities, free or cheap public transport, exercise facilities and classes, and funding. For example: “It's just a matter of council sort of supporting these things by making sure there is publicity for them and they've got the occasional sort of grant money or something.” CAU005.

## Discussion

This study examined perceived benefits of and barriers and enablers to PA in older Caucasian and Chinese adults living in Australia. Most participants reported knowing the benefits of PA for physical health and some understood its benefits for mental and cognitive health. However, many participants attributed the mental health benefits of PA to the opportunity to socialise with people rather than PA itself. Moreover, many participants were not sure or suspected whether PA could benefit cognitive health.

As described in the section Results and Table 2, participants from both ethnic groups reported several similar barriers and enablers to PA. However, older Chinese adults more often reported costs, lack of time due to caring for grandchildren, lack of motivation, distant exercise location, and lack of interest and environment as barriers. They also more often reported peer support or influence and pro-exercise culture as enablers. As expected, Chinese older adults reported some unique barriers relating to language and culture factors, and two unique enablers: unwilling to become a burden on their children and available PA opportunities. In comparison, older Caucasians more often reported self-motivation, enjoyment, maintaining a habit, previous exercise experience, having a fulfilling life, and the social aspects of exercise as enablers.

Participants' mixed views about the benefits of PA on mental health and cognitive health partially support the findings of previous research. One US study examining barriers and enablers to PA among several ethnic groups found that compared with physical health benefits, participants did not report mental health benefits of PA frequently (12). However, this study and another qualitative study, both from the *US Healthy Brain Initiative*, revealed that older adults believed in the cognitive health benefits of PA (4, 12). It is possible that in both studies older adults' positive views about the relationship between PA and cognitive health was influenced by the context - cognitive health - of the *Initiative*. A third US qualitative study involving older Black and White participants reported that older adults' understanding of the cognitive health benefits of PA was mixed, with some having previous knowledge, some needing more information, and some being sceptical (23).

In support of the literature, our findings suggest that older adults have inadequate knowledge about mental and cognitive health (3, 24). While both mental health and cognitive health include complex domains, participants in our study mostly related mental health to subjective well-being (feeling happier or good mood), and brain health to memory or brain thinking. There is a need to develop public messages or educational programs aimed at helping older adults enhance the knowledge of mental and cognitive health, and the mental and cognitive health benefits of PA. With such knowledge older adults are likely to feel more motivated to engage in PA (25).

The frequently reported barriers and enablers in our study are largely consistent with the findings of existing literature reviews focusing on older adults (1, 25). However, our study identified some novel barriers (e.g., older age and lack of interest.) and enablers (e.g., having a fulfilling life, having more time after retirement, and pro-exercise culture). We only identified one empirical study reporting that older age and disinterest were barriers to PA (26). More empirical research is needed to establish the evidence on these newly identified barriers and enablers to PA in older adults.

Consistent with the literature, we found that health issues operated as both barriers and enablers to PA (12, 25–27). Well-designed PA programs beneficial to the management of health issues, combined with educational messages about the health benefits of PA, will facilitate older adults to overcome health-related barriers to actively participate in PA (1). We also found that presence and absence of self-motivation, enjoyment, time, peer support and favourable environmental factors acted as enablers and barriers, respectively. PA programs with a focus on addressing these barriers might have great potential to effectively promote older adults to undertake and maintain PA (26).

Some factors, such as doing housework, childcare, bad weather and costs may not be barriers *per se*, but how older adults perceive them (21, 28). For example, both housework and caring for grandchildren may only be constraints for doing planned and organised exercise, whereas older adults can consider engaging in housework or childcare as PA fitting into their daily lives (27). Regarding bad weather and costs, there are alternative PA options such as indoor activities and walking that are deemed to be appropriate and beneficial for older adults (3, 27).

We identified notable differences in perceived barriers and enablers to PA between the two older ethnic groups, which could be related to the sample characteristics, context or acculturation [defined as a process of a person or group adopting certain values and practises of a new culture (29, 30). First, older Caucasians more often reported enablers and older Chinese adults more often reported barriers. These findings might reflect the fact that the older Caucasian participants in this study were physically more active, healthier and better educated than older Chinese adults (Table 1). Second, older Chinese adults frequently reported peer support as an enabler. This might indicate their needs for peer support due to feeling lonely or isolated by living in a foreign country (29). Third, older Chinese adults less frequently reported maintaining a habit, previous exercise experience, social aspects of exercise and having a fulfilling life as enablers. These findings could be related to older Chinese adults' low level of acculturation, leading to them having difficulty in maintaining an exercise routine, establishing social networks, doing things enjoyable, and enjoying lives in a new culture (30).

Special attention needs to be paid to some more common and unique barriers to PA for older Chinese adults such as lack of time due to childcare, lack of motivation, lack of interest, distant exercise location, costs, lack of fluency in English and feeling reserved (31). Community organisations with a focus on the Chinese culture can provide education sessions addressing older Chinese adults' language barriers and low motivation, organise group exercise, and provide transport support (32). Family members' support and encouragement and share of family duties could facilitate older Chinese adults to engage in more PA. Walking and free Tai Chi classes can be recommended as both are feasible, beneficial, culturally appropriate and also preferred types of PA for many older Chinese adults (3, 27, 30).

Our findings draw attention to barriers and enablers to PA at individual, health care system, community and government levels, suggesting that multi-level efforts are needed to effectively promote older adults to engage in and maintain PA (17). At the individual level, older adults can undertake activities that are enjoyable, less complex, and interactive with good social and natural environments. They can also make efforts to form a habit of doing exercise (1–3, 25). Older adults who lack confidence, do not do exercise very often or at all, and/or have health issues, may consider seeking professional advice to take up appropriate types and levels of PA (21).

The health care system, communities and governments have a pivotal role in promoting older adults to engage in and maintain PA. GPs are well-positioned to provide information on the multiple benefits of PA and available PA opportunities as part of their medical consultations. Meanwhile, medical or exercise specialists should prescribe exercise programs taking into account older adults' medical conditions and personal circumstances (31). Community organisations and local governments can advocate for PA by displaying PA information at community libraries and newsletters, as well as using mass media messages. They can also provide PA opportunities with low or no costs such as organising exercise groups and classes, providing spaces and exercise facilities, and implementing special exercise programs tailored to the needs of older adults from different ethnic groups (26, 27).

Lastly, we identified some barriers and enablers less frequently reported by older adults (Table 2). Notably, according to qualitative research, low frequency does not suggest that these barriers and enablers are not important (22). Given that we could not relate most of these less frequently reported barriers and enablers to previous literature (1, 25, 26), we suggest undertaking more research using qualitative, quantitative or mixed-research methods to confirm whether these are common factors influencing older adults to undertake and maintain PA.

### Strengths and Limitations

One strength of this study is the involvement of older Chinese participants with different levels of self-rated health and PA. Another strength is the integration of individual and group interviews with older Chinese adults, which allows for a more in-depth investigation of the study topic (27).

This study has some limitations. First, we found that most participants interpreted PA as a type of body movement that could be structured or unstructured, and gentle or vigorous, while a few participants did not regard low level of activities such as housework, gardening or slow walking as PA. Participants' different interpretations of PA may affect their views on the benefits of and barriers and enablers to PA. Second, older Caucasian and Chinese adults differed in self-reported health and PA level, and educational attainment. This makes it difficult to determine whether different views of the two ethnic groups on some barriers and enablers are associated with ethnicity or differences in sample characteristics (12). Third, most participants in both groups were women and lived in suburbs. Their views may not be well-generalised to older men and rural-dwelling older adults who have different exercise experiences and access to exercise facilities, community organisations, transportation and social networks (7). Future research should aim to involve those underrepresented participants to understand their perspectives.

Fourth, we transcribed and analysed group interviews at the level of individual interviews. This means that any quotes from the group interviews used to support our findings cannot be linked to individual participants. However, it was not our aim to examine whether specific participant characteristics (except for ethnicity, Chinese vs. Caucasian) affect their views on the study topic. Fifth, for logistic reasons, it was not possible for the two bilingual researchers to conduct all interviews together. However, the two researchers' using the same interview guide and exchanging ideas and strategies of conducting the interviews during this process have ensured a good level of consistency in the data collection. Lastly, only one researcher was involved in coding the data and coming up with the initial themes and subthemes. However, all co-authors interpreted these themes and subthemes until agreement was reached.

## Conclusions

In conclusion, our findings suggest that there are similarities and differences in perceived barriers and enablers to PA between older Caucasian and Chinese adults. In general, older Caucasians more often report enablers to PA while older Chinese adults more often report barriers to PA. In addition, older Chinese adults face language and culture related barriers to PA. While many barriers and enablers identified in our study can be related to previous literature, some newly identified barriers and enablers need to be confirmed by further research. Our findings about the multi-level barriers and enablers highlight that the community, health care system and governments all have an important role in promoting the uptake and maintenance of PA in the older population.

## Data Availability Statement

The datasets presented in this article are not readily available because the raw data are transcripts of individual interviews and group discussions, and without participants' consent these transcripts are not meant to be shared. Requests to access the datasets should be directed to Emily You *via* chuanmei.you@unimelb.edu.au.

## Ethics Statement

Ethical approval was obtained from the Human Research Ethics Committee of the University of Melbourne (Ethics ID: 1750807). We provided each participant with a plain language statement, project flyer and consent form written in English. We communicated the project information to participants in Mandarin or Cantonese verbally, if required. Most participants provided written consent while nine Chinese participants provided verbal consent for cultural reasons. The verbal consent was audio recorded at the beginning of the interview.

## Author Contributions

EY, KE, and NL designed the original research study. EY took the lead to conduct the research study and drafted the manuscript. KE and NL provided expert advice on undertaking the research study. CW made important contributions to undertaking the study, including data collection, and transcription. AG, EC, and TC assisted with participant recruitment and provided advice on undertaking the research study. All authors interpreted the findings (including reaching agreement on the themes and subthemes identified from the interview/focus group data). All co-authors NL, CW, AG, EC, TC, KA, FH, and KE provided important feedback.

## Funding

The authors wish to thank the funding support of the University of Melbourne Early Career Researcher Grant Scheme (502642), and the support of the Centre of Research Excellence in Cognitive Health (1100579) from the Australian National Health and Medical Research Council.

## Conflict of Interest

The authors declare that the research was conducted in the absence of any commercial or financial relationships that could be construed as a potential conflict of interest.

## Publisher's Note

All claims expressed in this article are solely those of the authors and do not necessarily represent those of their affiliated organizations, or those of the publisher, the editors and the reviewers. Any product that may be evaluated in this article, or claim that may be made by its manufacturer, is not guaranteed or endorsed by the publisher.
