# Supplementary material for: Ethnic Differences in Barriers and Enablers to Physical Activity Among Older Adults
Source: Front Public Health. 2021 Sep 10;9:691851. doi: 10.3389/fpubh.2021.691851 (PMC8460870; doi:10.3389/fpubh.2021.691851)
Supplement: Supplementary file 1 [file Table_1.DOCX]

# Interview Guide for Individual or Group Interview

1. What is your understanding of physical activity or exercise (definition, benefits, harms etc.)?

2. What kind of physical activity/exercise do you do (past and present)?

3. Are there certain types of physical activity/exercise that you do/do not enjoy? Are there any certain formats of physical activity/exercise that you prefer?

4. Are there any factors that facilitate or motivate you to undertake physical activity/exercise?

5. Are there any factors that make it difficult for you to undertake physical activity/exercise?

6. Are there any factors that prevent you from undertaking physical activity/exercise?

7. What do you think will make it easier for you to undertake physical activity/exercise more?

8. What can we do to help you to undertake physical activity/ exercise more?

9. Before we conclude the discussion, is there anything else relating to the study topic that you would like to discuss?
